# Ligand-assisted structure tailoring of highly luminescent Cu-In-Zn-S/ZnS//ZnS quantum dots for bright and stable light-emitting diodes

**DOI:** 10.3389/fchem.2022.1102514

**Published:** 2022-12-13

**Authors:** Shuaipu Zang, Xuhui Zhang, Yingying Sun, Ning Li, Lei Wang, Lin Song Li

**Affiliations:** ^1^ Key Laboratory for Special Functional Materials of Ministry of Education, National & Local Joint Engineering Research Centre for High-efficiency Display and Lighting Technology, School of Materials, Henan University, Kaifeng, China; ^2^ College of Science, Zhongyuan University of Technology, Zhengzhou, China

**Keywords:** Cu-In-Zn-S QDs, structure tailoring, 2-ethylhexanoic acid, alloyed interface layer, efficiency roll-off

## Abstract

Harnessing environment-friendly and low-cost multinary Cu-In-Zn-S quantum dots (QDs) as emitters for light-emitting diodes (LEDs) has attracted great attention for display and lighting application. However, suboptimal QD structure is a huge obstacle, which results in serious non-radiative recombination and efficiency roll-off. Herein, we synthesized structure-tailored Cu-In-Zn-S/ZnS//ZnS QDs by improving the reactivity of shell growth by 2-ethylhexanoic acid (EHA) ligands. The EHA-assisted shell growth can boost an extended alloyed layer at the core-shell interface and a smoothed confinement barrier, which effectively passivate the interface defects and suppress Förster resonance energy transfer (FRET) process. These synthesized QDs display a bright photoluminescence emission (quantum yield of 83%) and a larger size of 8.4 nm. Moreover, the resulting LEDs based on the EHA-assisted QDs exhibit a maximum luminance of 8074 cd/m^2^, and a current efficiency of 7.3 cd/A with a low efficiency roll-off. Our results highlight a remarkable ligand strategy to tailor the QD structure for high performance QD-based LEDs.

## 1 Introduction

Quantum dots (QDs) are developing dramatically for display and lighting application due to their remarkable optical and electrical properties such as high photoluminescence quantum yield, narrow emission linewidths, size-tunable emission wavelength and low-cost solution fabrication (Dai et al., 2014; Shu et al., 2020; Garcia de Arquer et al., 2021). QD-based light-emitting diodes (QLEDs) as superior light sources have attracted huge interest, since the first device was reported (Colvin et al., 1994; Deng et al., 2020). The high external quantum efficiency (EQE) and luminance have been achieved in red, green and blue QLEDs through the advances of device structure, QD material synthesis and surface ligand (Qian et al., 2011; Li et al., 2018a; Shen et al., 2019; Xu et al., 2020; Cheng et al., 2022; Lee et al., 2022; Meng et al., 2022). Yet almost all of the high performance QLEDs employed the Cd-based QDs as the emitting layer, which was limited by the “Restriction of Hazardous Substances” directive of the European Union. Therefore, the development of non-Cd QDs is urgent and crucial for QLED commercial application. In multi-type non-Cd visible QDs, Cu-In-Zn-S QDs exhibit a broad emission linewidths and tunable coverage green-red emission wavelength, which is regard as the excellent candidates for white-light illumination and outdoor signal sources (De Trizio et al., 2012; Zhang et al., 2013; Chen et al., 2018).

The first Cu-In-Zn-S based QLEDs were built by the mixture of red Cu-In-Zn-S/ZnS QDs and blue-green polymer in 2011, which exhibited a luminescence of 450 cd/m^2^ and a high color rendering index of 92 (Zhang et al., 2011). Whereafter, enormous advances were gained toward the high-performance Cu-In-Zn-S based QLEDs in terms of the QD optimization of surface ligand engineering and core/shell nanostructure (Tan et al., 2011; Park et al., 2015; Bai et al., 2016; Wang et al., 2018; Li et al., 2020; Ye et al., 2020). For example, Tan et al. synthesized red, yellow and green Cu-In-Zn-S/ZnSe/ZnS core/shell QDs (diameters: 2.3, 2.7, and 3.3 nm, respectively), and further fabricated electroluminescent devices with a maximum brightness of 1200–1600 cd/m^2^ (Tan et al., 2011). Short-chain 6-mercaptohexanol and 1,2-ethanedithiol ligands were treated on the QD surface to efficiently promote the carrier inject balance, produced an EQE of 3.22% and a maximum luminance of 8,735 cd/m^2^ (Bai et al., 2016). Nevertheless, serious essential issues about Cu-based QD materials still impede the performance improvement of electroluminescent (EL) QLEDs. Firstly, stoichiometry control of Cu-based cores is difficult due to the huge reactivity difference of Cu and In precursors. Cu/In molar ratio, solvent and surface ligands were systematacially researched to optimize the crystal structure and optical properties of Cu-In-Zn-S cores (Li et al., 2011; Song and Yang, 2012). Secondly, large lattice mismatch between Cu-based cores and ZnS shells results to serious interface defects and suboptimal photoluminescence quantum yields (QY). During the ZnS shell growth, Zn anion gradually incorporates into Cu-In-S cores, which leads to the significant improvement of QY (Berends et al., 2018). In addition, we reported a facile and reliable non-injection method to synthesis Cu-In-Zn-S/ZnS core shell QDs with a QY of 60% and a tunable photoluminescent in the range 580–780 nm (Shen et al., 2012). Bawendi group investigated the effects of Zn precursors (Zn-carboxylate and Zn-thiolate) on the ZnS shell growth. Zn-carboxylate precursors could promote the interfacial cation-exchange process to generate the alloyed shell, which significantly contributed to the QY improvement (Hansen et al., 2019). Recently, a low-temperature hot injection method using a high-reactive sulfur source (S powder dispersed in oleylamine) was reported to synthesized Cu-In-Zn-S/ZnS QDs with an alloyed interface, leading to high QY of 85% at 530 nm. As a result, the EL devices obtained a maximum luminance of 4450 cd/m^2^ and current efficiency of 3.52 cd/A (Deng et al., 2021). Thirdly, serious Förster resonance energy transfer (FRET) process results to tremendous carrier non-radiative recombination and efficiency roll-off in the devices due to the small QD size. The double-shelled strategy was introduced to synthesize Cu-In-S/ZnS//ZnS QDs with a size of 4.3 nm and a QY of 80%, which shown excellent properties in the two-band white LEDs (Park et al., 2015). Afterwards, Kim *et al.* further developed shell growth process with a long duration and synthesized Cu-In-S based QDs with a QY of 89% and size of 7.1 nm. The larger QD devices displayed a maximum luminance (L_max_) of 8464 cd/m^2^ and an EQE of 7.3% (Kim and Yang, 2016). However, the thicker ZnS shell could aggravate the carrier injection unbalance and lead to the efficiency roll-off in the device (Li et al., 2018b; Cao et al., 2018). Therefore, high-quality Cu-In-Zn-S based QDs with tailored nanostructures not only need the large size, but also should increase the thickness of the alloy layer. How to promote the growth of the alloy layer is crucial and urgent for the chemical synthesis of high-quality Cu-In-Zn-S core/shell QDs.

Herein, we introduce a facile synthesis method of Cu-In-Zn-S/ZnS//ZnS QDs employing high reactivity zinc-octoate (the reaction product of ZnO and 2-ethylhexanoic acid (EHA) at 150°C) as the Zn precursor during the outer ZnS growth process. The EHA-activated Cu-In-Zn-S/ZnS//ZnS QDs reach a high QY of 83%, and a larger average size of 8.5 nm. The extended alloyed interface layer can effectively suppress Förster resonance energy transfer (FRET) process and smooth confinement barrier. The QLEDs based on the EHA-activated QDs exhibit a maximum luminance of 8074 cd/m^2^, a peak EQE of 1.9%, and a current efficiency of 7.3 cd/A with a low efficiency roll-off. Our works forcefully indicate that ligand manipulation during the shell growth can effectively tailor the QD structure for the improvement of the EL devices.

## 2 Materials and methods

### 2.1 Materials

Copper (I) iodide (CuI, 99.99%), indium acetate (In (AC)_3_, 99.99%), zinc acetate (Zn (ac)_2_, 99.99%), 1-octadecence (ODE, 90%), 1-octanethiol (OT, 98%), zinc stearate (Zn-St, 99.99%), oleic acid (OA, 90%) and 2-Ethylhexanoic acid (EHA, 99%) were purchased from Sigma-Aldrich. Octane, ethanol and acetone were purchased from Aladdin. All the chemicals were used without further purification.

### 2.2 Synthesis of O-QDs and E-QDs

Zn-St precursor (0.5 M): 12 mmol of Zn-St, 4 ml of OA, and 20 ml of ODE were mixed in a three-neck flask and heated at 150°C for 30 min to obtain a clear solution. Zn-OA precursor (0.5 M): 15 mmol of Zn (ac)_2_, 15 ml of OA, and 15 ml of ODE were mixed and heated at 120°C for 60 min to obtain a clear solution. Zn-EHA precursor (0.5 M): 15 mmol of Zn (ac)_2_, 15 ml of EHA, and 15 ml of ODE were mixed and heated at 100°C for 30 min to obtain a clear solution. All the flask should be degassed under vacuum at 100°C for 10 min to remove the water gas.

In a typical procedure, CuI (0.0245 g), In (ac)_3_ (0.143 g) and Zn (ac)_2_ (0.1101 g) were mixed with 5 ml of ODE in a 25 ml three-necked flask. The mixture was heated to 230°C under magnetic stirring and nitrogen flow for 15 min. Then, 5 ml of OT was quickly injected into the system. The mixed solution was kept at 230°C for 10 min to the core growth. Without further purification, 12 ml of Zn-St precursor was added dropwise at a rate of 4 ml/h for the inner ZnS shell growth. For the O-QDs, Zn-OA precursor was added dropwise at a rate of 4 ml/h for the outer ZnS shell growth. For the E-QDs, Zn-OA solution was replaced by Zn-EHA precursor to the formation of the outer ZnS shell with the extended alloyed interface layer. The synthesized O-QDs and E-QDs were purified by ethanol and acetone for further characterization and device fabrication.

### 2.3 Device fabrication

The pre-patterned indium tin oxide (ITO) glass substrates were thoroughly washed three times including deionized water, acetone and isopropyl alcohol, then treated by UV-ozone for 15 min. Next, PEDOT:PSS solution was spin-coated on the substrates at 5500 rpm for 30 s and annealed at 150 °C for 15 min. Then, these substrates were transferred to a N_2_-filled glovebox to the next step. Subsequently, the TFB layers were deposited by spin-coating the TFB in chlorobenzene solution (8 mg/ml) at 3000 rpm for 30 s and annealing at 150°C for 30 min. The QD emitting layers were deposited by spin-coating the QD in n-octane solution (18 mg/ml) on the TFB layer at 2000 rpm for 30 s. And after 20 min, the ZnO layer were deposited by spin-coating the ZnO nanoparticle in ethanol solution (40 mg/ml) at 2000 rpm for 30 s and annealing at 60°C, 30 min. Finally, the films were loaded into a vacuum chamber to deposit Al cathode (100 nm, vacuum pressure ≈3 × 10^−7^ torr) and encapsulated by UV-curable resin with a cover glass. The effective area of the device is 4.0 mm^2^ by a shadow mask.

### 2.4 Characterization

The absorption spectra were measured by a Lambda 950 PerkinElmer spectrometer. And the PL spectra were performed using a HORIBA FluoroLog-3 spectrofluorometer. All QY data was obtained by Ocean Optics USB2000 spectrometer, equipped with Ocean Optics ISP-50-8-1 integrating sphere. PL lifetime spectra were obtained by a JY HORIBA FluoroLog-3 fluorescence spectrometer equipped with a 405 nm picos pulsed diode laser with 1 MHz repetition rate and ∼200 ps pulse duration. All the QD samples were diluted to be in a range of 0.02–0.05 of optical density values. Transmission electron microscope (TEM) images were performed by JEOL JEM-2100. X-ray diffraction (XRD) pattern were measured by Bruker D8 Advance with Cu-Kα radiation. FTIR spectra and mass spectra were measured by BLUKER INVENIOS and BLUKER SolariXTMXR, respectively. The current density-voltage-luminance curves were obtained by a Keithley 2400 source meter, equipped with a Keithley 6485 picoammeter coupled with a Si photodetector (Newport 818-UV) under ambient conditions. The luminance was collected by a Photo Research 735 (PR-735) spectrometer.

## 3 Results and discussion

**FIGURE 1:**
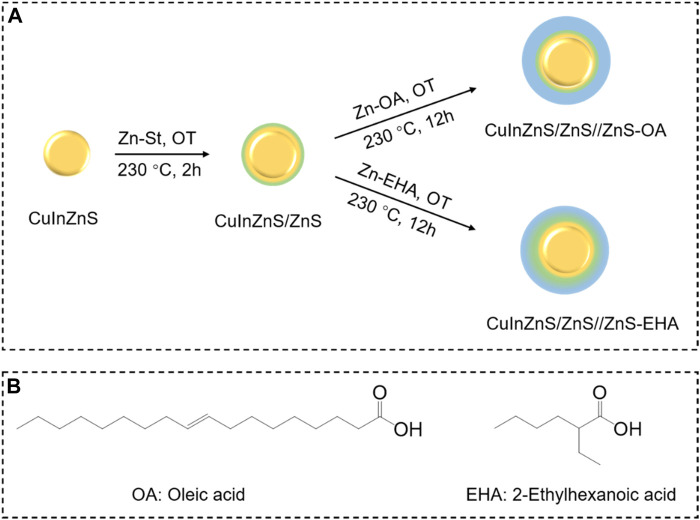
**(A)** Schematic illustration of the synthesis route for CuInZnS/ZnS//ZnS QDs with OA (up route) and EHA (down route) strategy; **(B)** Molecular structures of oleic acid and 2-ethylhexanoic acid.

The synthetic route of Cu-In-Zn-S core/shell QDs are illustrated in Figure 1A, in which the down route is the novel EHA-assisted strategy, and the traditional OA-assisted method (the up route) is also exhibited for comparison. In a typical Cu-In-Zn-S core-shell QD synthetic process, Cu-In-Zn-S cores are prepared by the hot-injection method. Zinc stearate (Zn-St) and 1-octanethiol (OT) are employed as precursors for the growth of the inner alloyed ZnS layer. For the EHA-assisted strategy, Zn-EHA and OT are used as precursors at 230 °C for the growth of the outer alloyed ZnS shell. As comparison, zinc oleate (Zn-OA) and OT are used as precursors to grow the pure ZnS shell. As shown in Figure 1B, EHA has a shorter chain length, which makes Zn-EHA precursor possess a small steric hindrance compared with Zn-OA precursor. In addition, the dissociation constant (pK_a_) of EHA (4.895, 25°C) is less than that of oleic acid (5.35, 25°C), which means that Zn-EHA precursor has higher reactivity and can effectively promote the formation of alloyed layer.

**FIGURE 2:**
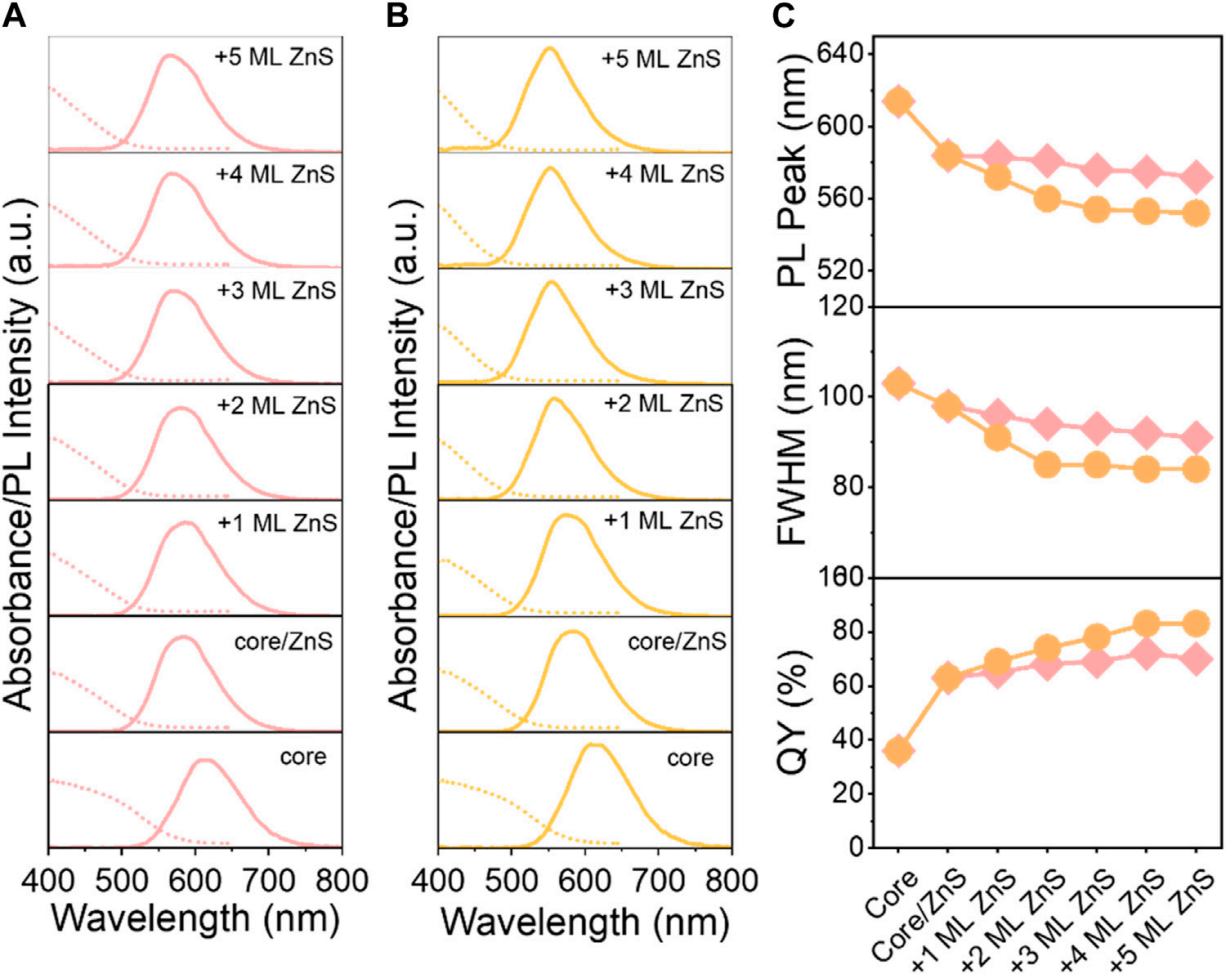
**(A,B)** Evolution of absorption and PL spectra; **(C)** PL peak position (top), FHWM (middle) and QY (bottom) of O-QDs (pink lines) and E-QDs (orange lines) upon shell growth.

In order to study the influence of EHA ligands on the outer ZnS shell, we systematically measured the evolution of absorption fluorescence with the shell growth (Figures 2A,B). OA-assisted Cu-In-Zn-S/ZnS//ZnS core/shell QDs, namely O-QDs, were synthesized based on our previous work (Wu et al., 2018), and EHA-assisted Cu-In-Zn-S/ZnS//ZnS core/shell QDs were named E-QDs for short. As a consequence of growth of the inner ZnS shell onto Cu-In-Zn-S cores, their absorption and PL peaks are blue-shifted obviously, which is attributed to the core-shell interface alloying due to Zn diffusion (Figure 2C). (Park and Kim, 2011; Liu et al., 2020) With the growth of a 5 monolayer (ML) outer ZnS shell, the absorption and PL peaks of the two QDs continue to blue-shift, and E-QDs have a greater degree of blue shift than O-QDs. The PL full width at half-maximum (FWHM) of E-QDs decreases gradually from 98 nm (0 ML of the outer ZnS) to 84 nm (0 Ml of the outer ZnS) and that of O-QDs decreases from 98 nm to 91 nm. Meanwhile, the QY of E-QDs improves significantly, which increases to 83% of E-QDs with 5 Ml of outer ZnS shell from 63% of E-QDs without the outer ZnS shell. As a contrast, the O-QDs with 5 ML of outer ZnS shell represent a QY of 70%. The improved QY of E-QDs is attributed to the higher reactivity of the EHA ligands, which can accelerate the growth of alloyed layer to eliminate the defects in the core-shell interface. As a result, the larger blue-shift, narrower FWHM and higher QY improvement demonstrates that EHA ligands can effectively confine excitons and passivate the defects of the core-shell interface due to the extended alloyed layer.

**FIGURE 3:**
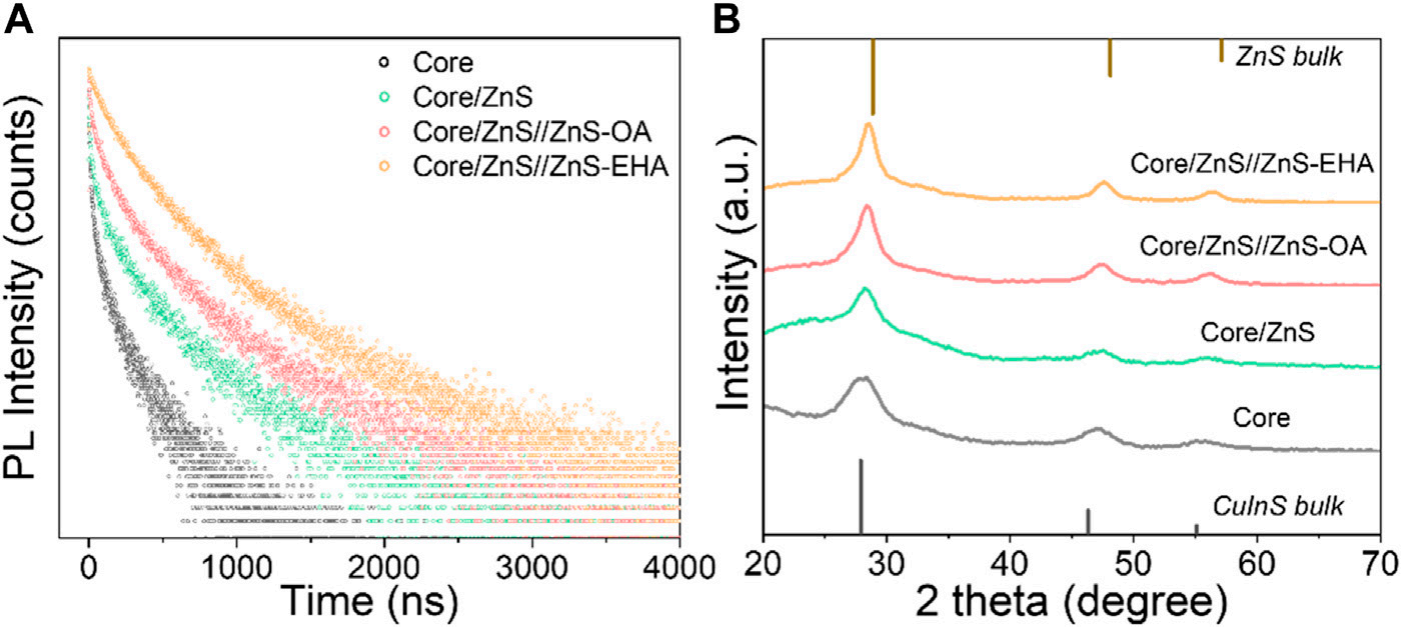
Evolution of PL decay dynamics **(A)** and XRD patterns **(B)** for O-QDs and E-QDs upon shell growth.

**FIGURE 4:**
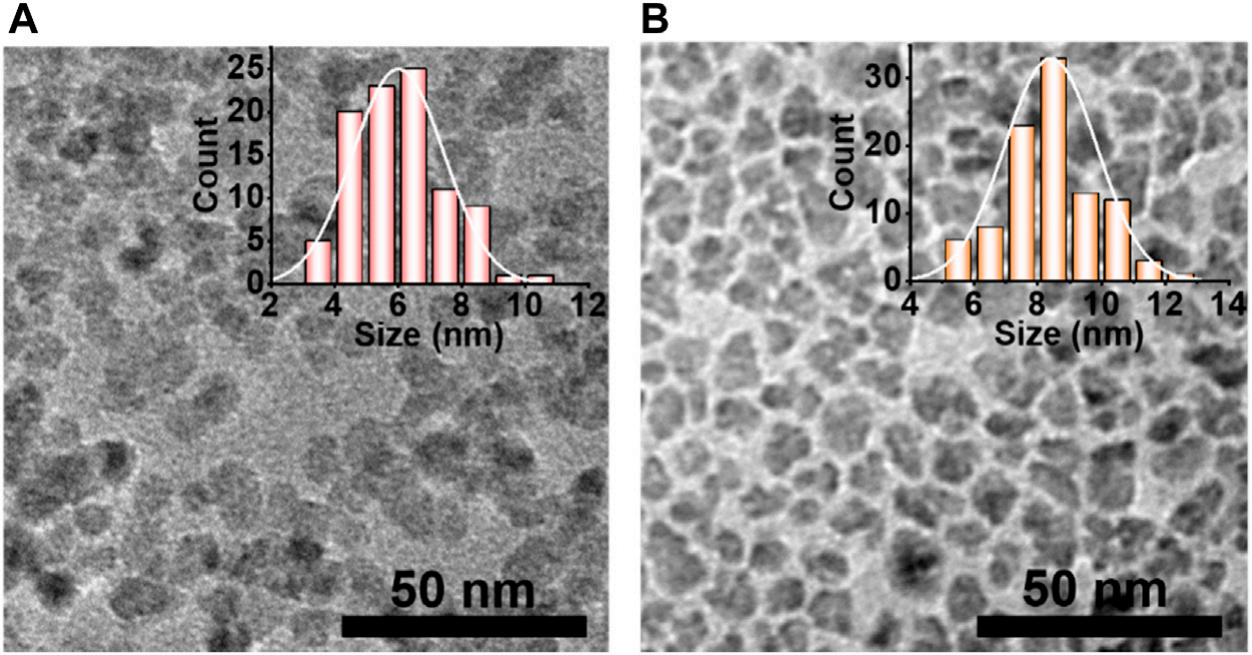
TEM images of **(A)** O-QDs and **(B)** E-QDs; Inset: corresponding size distribution histograms.

The evolution of PL decay dynamics (Figure 3A) for O-QDs and E-QDs upon the shell growth were carried out to further understand the mechanism of the PL emissions for the EHA-assisted shell growth. All the samples represent a triexponential decay way, suggesting the different recombination channels of excitons (Zhong et al., 2008; Huang et al., 2017). PL lifetimes (τ) and amplitude constant ratios (A) by fitting were extracted in Table 1. The short lifetime τ_1_ was assigned to the surface defect-related non-radiative recombination and the intermediate lifetime τ_2_ was attributed to the interface-related donor-acceptor pair recombination. In addition, the long lifetime τ_3_ was associated with the core-related donor-acceptor pair transitions. In comparison with Cu-In-Zn-S core, a smaller A_1_ indicates the less surface defects in Cu-In-Zn-S/ZnS QDs, which can ascribe to the inner ZnS shell growth. It is worth noting that the PL lifetimes τ_2_ about the interface-related donor-acceptor pair greatly increases after the outer ZnS growth, especially for E-QDs implying the extended alloyed interface layer with a smoothed confinement barrier. The average lifetime (τ_average_, 526.6 ns) of E-QDs is larger than that (424.4 ns) of O-QDs, which demonstrates the increased PL lifetime due to the incorporation of EHA ligands during the outer ZnS shell growth. X-ray diffraction (XRD) patterns were measured to explored the structure evolution of E-QDs (Figure 3B). With the shell growth, the crystal structure of QDs is always maintained as a tetragonal structure. Furthermore, the diffraction peaks become narrower and moved gradually toward higher angles, which indicates that the formation of ZnS shell and the increased QD size. Since both OA and EHA ligands have the−CH_3_,−CH_2_-,−COO- groups, the Fourier transform infrared (FTIR) signal peaks of the two QDs are basically the same (Supplementary Figure S1). Mass spectrum of E-QDs contains an obvious signal peak of 144.56 g/mol, which indicates that EHA ligands are adsorbed on the QD surface (Supplementary Figure S2). Figure 4 shows the transmission electron microscopy (TEM) images of O-QDs and E-QDs, where the average size are 8.47 ± 1.6 nm and 5.99 ± 1.4 nm, respectively. The enlarged size of E-QDs means that Förster resonance energy transfer (FRET) can effectively suppressed in the QLEDs (Li et al., 2018b). Meanwhile, the E-QD film reveals a PL QY of 68%, which is a slight drop from that (83%) of E-QD solution (Supplementary Figure S3). Whereas, the PL QY (49%) of the O-QD film decreased obviously compared with that (70%) of O-QD solution. This suggests that the FRET process in the E-QDs is significantly inhibited due to the increased size. According to literatures, we speculated that larger E-QDs with the improved alloyed interface was the excellent candidate for the EL device, which could prominently restrain Auger-related non-radiative recombination process and luminescence quenching (Li et al., 2018b; Liu et al., 2020; Lee et al., 2022).

**TABLE 1:** PL lifetimes and amplitude constant ratios of O-QDs and E-QDs upon shell growth.

Sample	τ_1_/ns	A_1_/%	τ_2_/ns	A_2_/%	τ_3_/ns	A_3_/%	τ_average_/ns
Core	2.2	64.7	22.6	26.4	214.0	8.9	159.0
Core/ZnS	2.3	59.0	46.2	26.8	413.2	14.1	342.6
Core/ZnS//ZnS-OA	8.8	41.8	109.3	39.1	563.8	19.1	424.4
Core/ZnS//ZnS-EHA	67.8	24.7	281.7	60.4	897.4	14.8	526.6

**FIGURE 5:**
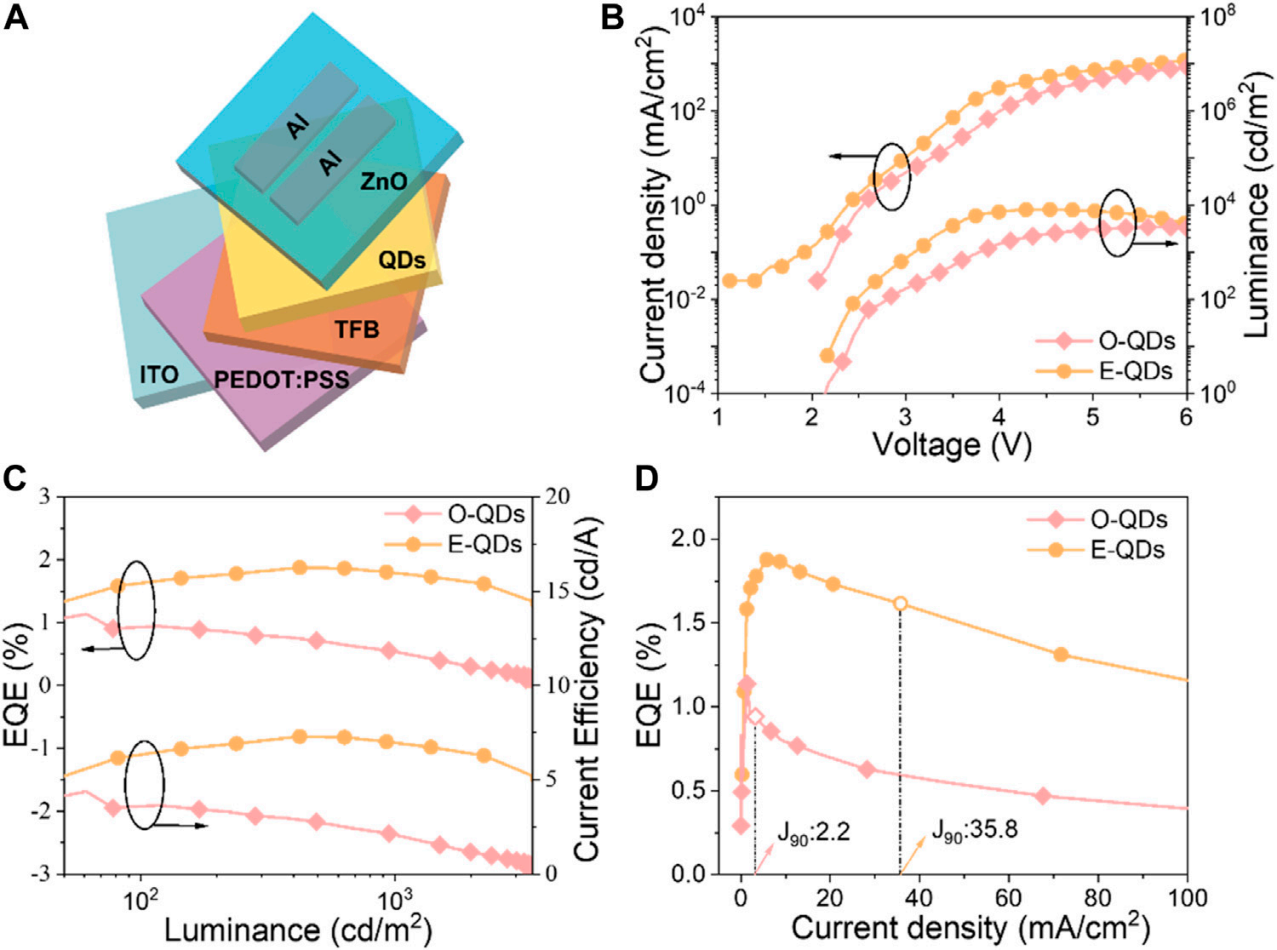
**(A)** Schematic device structure of the QLEDs; **(B)** Current density–Luminance–Voltage characteristics curves; **(C)** EQE–current efficiency–luminance characteristics; **(D)** Comparison of EQE−luminance characteristics for the devices based on the O-QDs and E-QDs.

To investigated the device performance of O-QDs and E-QDs, the QLEDs were fabricated with the device structure of indium tin oxide (ITO)/PEDOT:PSS/poly (9,9-dioctylfluorene-co-N-(4-(3-methylpropyl))diphenylamine) (TFB)/QDs/ZnO/Al (Figure 5A). Figure 5B shows the voltage dependent variations of current density and luminance of the two QLEDs for O-QDs and E-QDs with PL peaks at 551 nm and 566 nm, respectively. The E-QD based device reveals the higher current density and luminance compared with the O-QD device. The EL peaks of the two devices exhibit slightly red-shift compared to these corresponding PL peaks (Supplementary Figure S4), and the photographs of the EL devices operating at 7 V show a bright luminescence in Supplementary Figure S5. The E-QD device displays a larger L_max_ of 8074 cd/m^2^ and a peak current efficiency of 7.3 cd/A than those (3428 cd/m^2^ and 4.4 cd/A) of the O-QD device. The peak EQE of the O-QD and E-QD device are 1.1% and 1.9%, respectively (Figure 5C; Supplementary Table S1). The EQE value is lower than the record value in the references (Wang et al., 2018), which may be attributed to the unoptimized device structure. Remarkably, a low efficiency roll-off is proved by the EQE of the E-QD device at least 1.5% during the range of device luminescence from 80 to 2000 cd/m^2^. As contrast, the EQE of the O-QD device sharply drops down from the peak EQE (1.1%) at 60 cd/m^2^ to 0.3% at 2000 cd/m^2^. To further analyze the efficiency droop, a critical parameter J_90_ (the current density when the EQE dropped to 90% of its peak value) was extracted from EQE-luminance curves in Figure 5D. The J_90_ value (35.8 mA/cm^2^) of the E-QD device is 16 times larger than that (2.2 mA/cm^2^) of the O-QD device, which indicates that the efficiency roll-off is more slowly in the E-QD device. The improved performance and suppressed efficiency roll-off of the E-QD QLED are attributed to the increased QY, suppressed FRET and smoothed confinement barrier due to the extended alloyed interface of E-QDs. The E-QD devices with high luminance and low efficiency roll-off are not only expected to be used in lighting and display, but also desired as a potential candidate in other applications such as signal indications, lithography labs, and fog lights.

## 4 Conclusion

In summary, we introduced a ligand-assisted shell growth strategy for high quality Cu-In-Zn-S/ZnS//ZnS QDs, during which the EHA ligands with high reactivity played a crucial role in the formation of alloyed interface layer. The synthesized EHA-assisted QDs reveal a high QY of 83% and a large size of 8.4 nm. The extended alloyed layer can dramatically passivate the interface defects, smooth the confinement barrier, and suppress FRET. As a result, The QLEDs with EHA-assisted QDs exhibit a high EQE of 1.9%, luminance of over 8000 cd/m^2^ and a low efficiency roll-off. This work provides a feasible path to tailor QD structures for high performance non-Cd QLEDs.

## Data Availability

The original contributions presented in the study are included in the article/Supplementary Material, further inquiries can be directed to the corresponding authors.
