# Supplementary material for: Ligand-assisted structure tailoring of highly luminescent Cu-In-Zn-S/ZnS//ZnS quantum dots for bright and stable light-emitting diodes
Source: Front Chem. 2022 Dec 13;10:1102514. doi: 10.3389/fchem.2022.1102514 (PMC9792774; doi:10.3389/fchem.2022.1102514)
Supplement: Supplementary file 1 [file DataSheet1.docx]

Supplementary Material


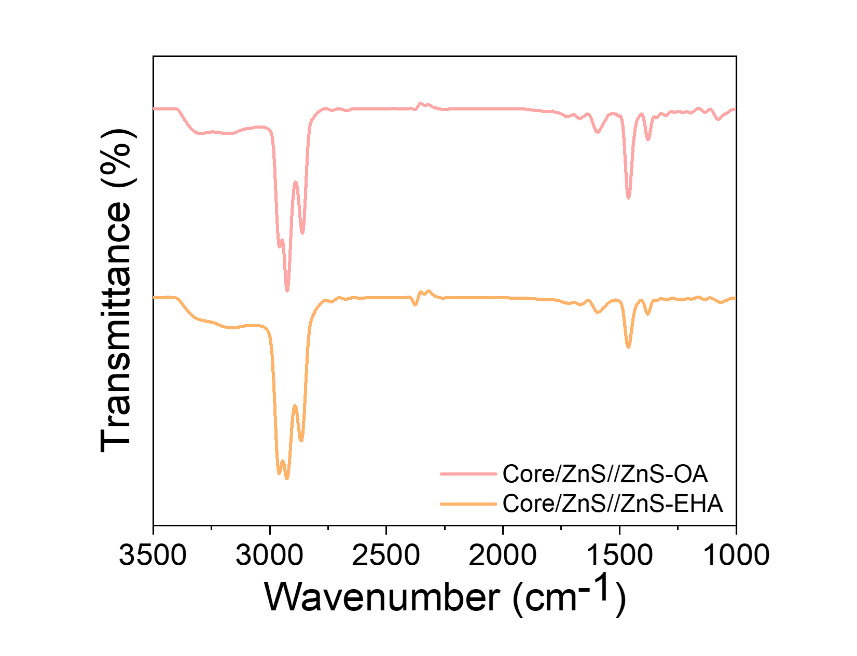


Supplementary Figure 1. FTIR spectra of O-QD and E-QDs.


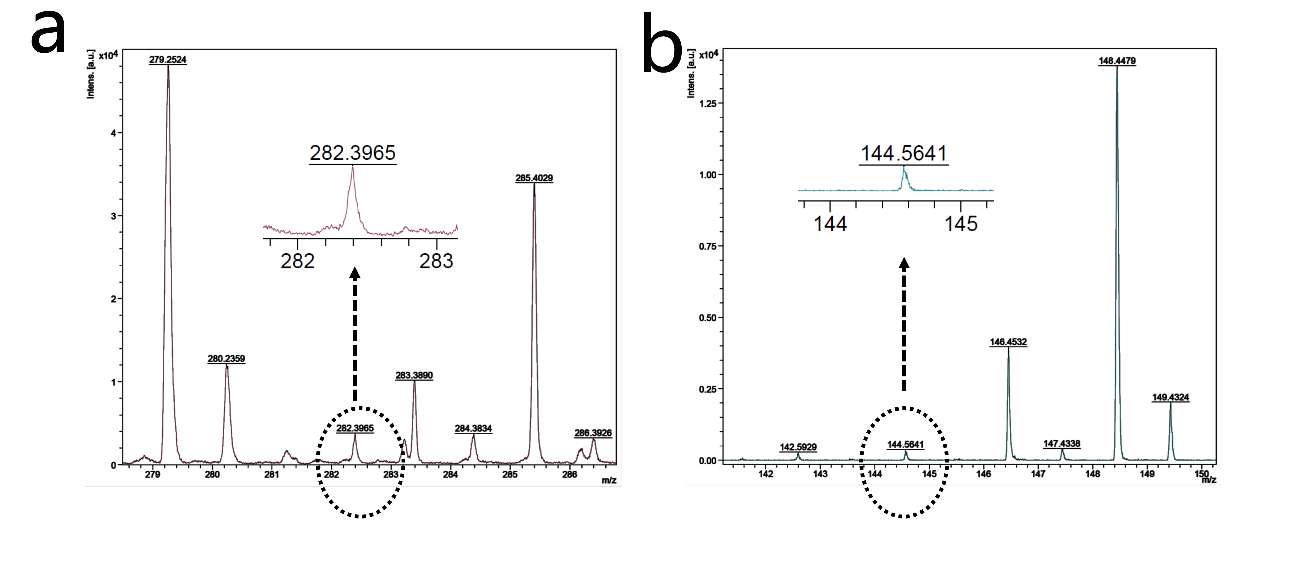


Supplementary Figure 2. Mass spectrum of O-QDs (a) and E-QDs (b).


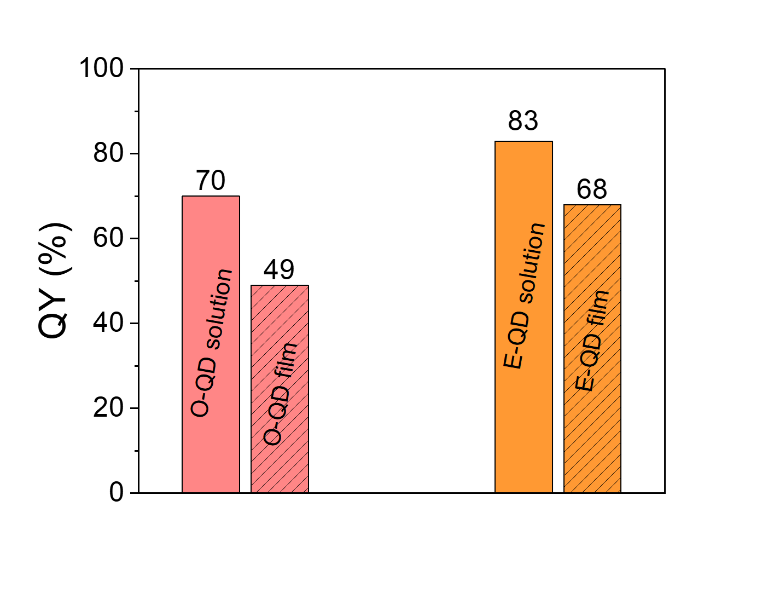


Supplementary Figure 3 QY of O-QDs and E-QDs in solution and film.


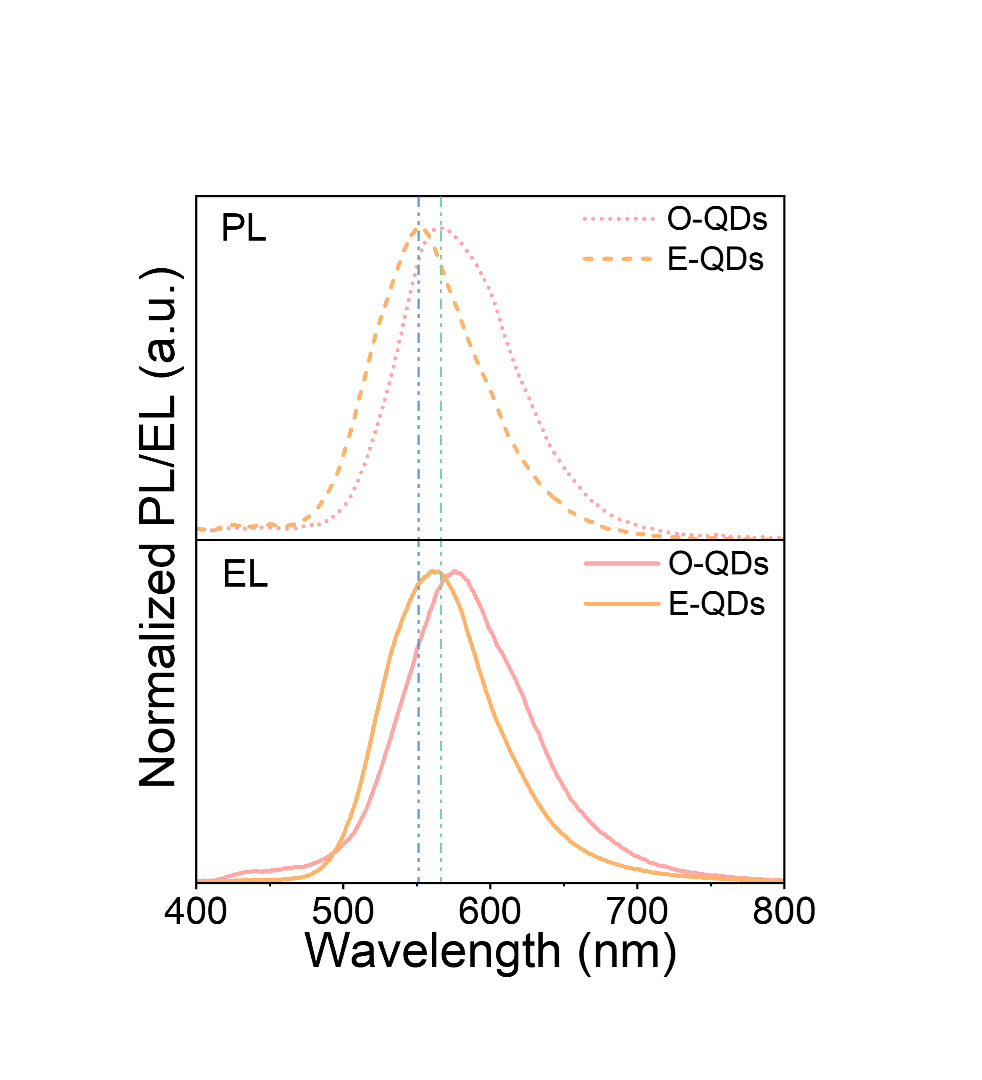


Supplementary Figure 4. Normalized PL spectra and EL spectra of QLEDs with O-QDs and E-QDs.


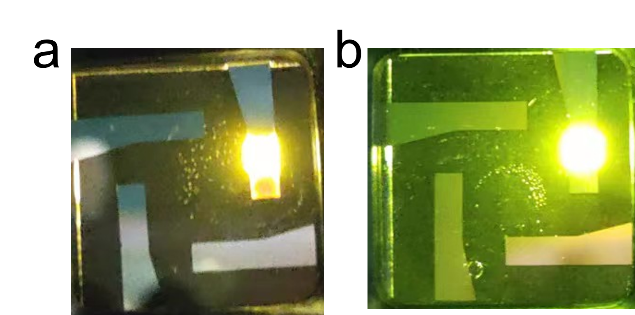


Supplementary Figure 5. Photographs of the EL devices with O-QDs (a) and E-QDs (b) operating at 7 V.

Supplementary Table 1. Summary of performance parameters of QLEDs based on O-QDs and E-QDs.

| Samples | L_max_ (cd/m^2^) | Peak EQE (%) | peak current efficiency （cd/A） | V_turn-on_ （V） |
| --- | --- | --- | --- | --- |
| O-QDs | 3428.3 | 1.1 | 4.4 | 2.1 |
| E-QDs | 8073.7 | 1.9 | 7.3 | 2.2 |
